# Complexities in Isolation and Purification of Multiple Viruses from Mixed Viral Infections: Viral Interference, Persistence and Exclusion

**DOI:** 10.1371/journal.pone.0156110

**Published:** 2016-05-26

**Authors:** Naveen Kumar, Sanjay Barua, Thachamvally Riyesh, Kundan K. Chaubey, Krishan Dutt Rawat, Nitin Khandelwal, Anil K. Mishra, Nitika Sharma, Surender S. Chandel, Shalini Sharma, Manoj K. Singh, Dinesh K. Sharma, Shoor V. Singh, Bhupendra N. Tripathi

**Affiliations:** 1 Division of Animal Health, ICAR-Central Institute for Research on Goats, Makhdoom, Mathura, India; 2 National Centre for Veterinary Type Culture Collections, ICAR-National Research Centre on Equines, Hisar, Haryana, India; 3 Department of Veterinary Physiology and Biochemistry, Lala Lajpat Rai University of Veterinary and Animal Sciences, Hisar, Haryana, India; University of California, Riverside, UNITED STATES

## Abstract

Successful purification of multiple viruses from mixed infections remains a challenge. In this study, we investigated peste des petits ruminants virus (PPRV) and foot-and-mouth disease virus (FMDV) mixed infection in goats. Rather than in a single cell type, cytopathic effect (CPE) of the virus was observed in cocultured Vero/BHK-21 cells at 6^th^ blind passage (BP). PPRV, but not FMDV could be purified from the virus mixture by plaque assay. Viral RNA (mixture) transfection in BHK-21 cells produced FMDV but not PPRV virions, a strategy which we have successfully employed for the first time to eliminate the negative-stranded RNA virus from the virus mixture. FMDV phenotypes, such as replication competent but noncytolytic, cytolytic but defective in plaque formation and, cytolytic but defective in both plaque formation and standard FMDV genome were observed respectively, at passage level BP8, BP15 and BP19 and hence complicated virus isolation in the cell culture system. Mixed infection was not found to induce any significant antigenic and genetic diversity in both PPRV and FMDV. Further, we for the first time demonstrated the viral interference between PPRV and FMDV. Prior transfection of PPRV RNA, but not Newcastle disease virus (NDV) and rotavirus RNA resulted in reduced FMDV replication in BHK-21 cells suggesting that the PPRV RNA-induced interference was specifically directed against FMDV. On long-term coinfection of some acute pathogenic viruses (all possible combinations of PPRV, FMDV, NDV and buffalopox virus) in Vero cells, in most cases, one of the coinfecting viruses was excluded at passage level 5 suggesting that the long-term coinfection may modify viral persistence. To the best of our knowledge, this is the first documented evidence describing a natural mixed infection of FMDV and PPRV. The study not only provides simple and reliable methodologies for isolation and purification of two epidemiologically and economically important groups of viruses, but could also help in establishing better guidelines for trading animals that could transmit further infections and epidemics in disease free nations.

## Introduction

Occurrence of multiple virus infections is ubiquitous in natural populations, which may have significant epidemiological and biological effects [1,2]. It is most commonly observed in immunocompromized individuals such as those infected with human immunodeficiency virus type 1 (HIV-1) [3]. Among the acute viruses, respiratory syncytial virus (RSV) and influenza virus infection in humans has been the most commonly reported mixed infection [3]. Peste des petits ruminants virus (PPRV) and orf virus (ORFV) [4], PPRV and blue tongue virus (BTV) [5], PPRV and other respiratory viruses [6], PPRV and goatpox virus [7], PPRV and Border disease virus [8], PPRV, BTV, rinderpest, and Rift Valley fever virus [9], are some of the mixed infections that have been observed in animals. The evidences of multiple virus infection in most of the above studies were determined by non-culture methods (serology/genome), however, isolation and purification of virus, particularly more than one virus, has not been well documented.

PPRV is a negative stranded RNA virus that belongs to the genus Morbillivirus of the family *Paramyxoviridae* [10,11], whereas foot-and-mouth disease virus (FMDV) is a positive stranded RNA virus that belongs to the genus Apthovirus under the family *Picornaviridae* [12]. PPRV is grouped into four genetic lineages (lineage I-IV) but a cross protection is believed to occur among PPRV strains from all different lineages. However, FMDV has seven distinct serotypes (O, A, C, Asia-1, SAT-1, SAT-2 and SAT-3) and multiple subtypes, and cross protection does not occur even within subtypes of a particular serotype [12,13]. Both PPRV and FMDV cause an acute contagious infection that results in significant economic losses to the livestock industry [14,15] and hence both are classified as Office International des Epizootics (OIE)-listed disease. Clinical signs of both the diseases mimic and are characterized by fever, erosive lesions on mucous membranes of oral cavity and, salivary and nasal discharge which are difficult to differentiate clinically. Except young animals which may die due to myocarditis, FMDV infected animals usually recovers after an acute infection without any significant mortality. FMDV causes a mild disease in small ruminants, which may not be clinically apparent [12,16]. Despite representing largest part of the world’s FMDV-susceptible domestic livestock, sheep and goats have generally been neglected with regard to their epidemiological role [16]. Nevertheless, both sheep and goats may become FMDV carriers and hence act as reservoirs for further infection and spread of the disease. Trade of live sheep and goats therefore, presents a major risk of entry of the FMDV to the disease-free countries [16]. PPRV infection on the other hand leads to high morbidity and mortality in small ruminants [17–19] with a sub-clinical infection in cattle and buffaloes [14].

Based upon the infection time, mixed infection is classified as coinfection when both the viruses infect simultaneously or, superinfection when one virus invades the host prior to the second virus [20]. Viral interference is a phenomenon whereby one virus inhibits replication of other viruses. If both the viruses belong to the same family, the interference is referred to as homologous viral interference [21], whereas within the same species but different serotype, it is referred to heterotypic interference [22]. Among viruses of different families, it is referred to as heterologous interference [20]. The coinfection may result in either coexistence (viral accommodation) of both the viruses or elimination (virus exclusion) [23] of one and survival of another (persistence). The epidemiology of PPR and FMD very much overlaps, and currently both are endemic in most parts of Africa and Asia [14,24–27] suggesting mixed infection of FMDV and PPRV may occur. Detection and purification of multiple viruses from mixed infections represents a major bottleneck in such epidemiological studies and thus needs to be established.

## Results

### Isolation and identification of the agents

The affected goats predominantly exhibited lesions on the tongue and gums, lameness due to swelling of the interdigital space (few animals), lacrymation (few animals), salivation, mucopurulent nasal discharge, dyspnea, diarrhea (few animals) and death with a mortality rate of 51.63%. The clinical signs were suggestive of a mixed infection of PPRV and FMDV.

**Fig 1:**
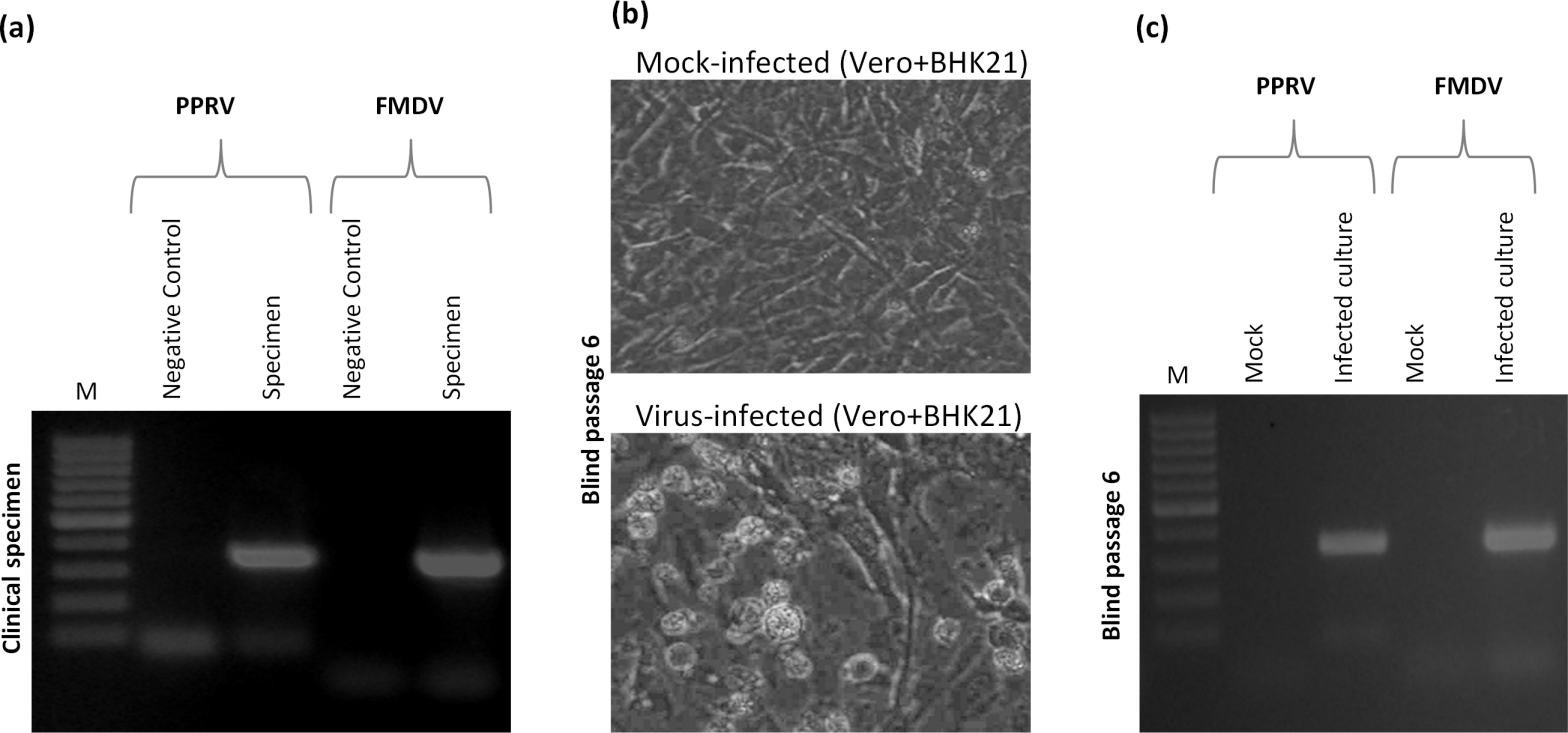
Detection and isolation of PPRV and FMDV from the clinical specimens. (a) Detection of PPRV and FMDV-specific genomes from the clinical specimens: Virus(es) recovered from the clinical specimen along with a negative control (MEM only) were tested for FMDV and PPRV-specific gene segments by PCR. (b) Virus isolation: Confluent monolayers of cocultured BHK-21/Vero cells were infected with virus recovered from a single specimen (salivary discharge) for 6 hours (h) followed by washing with PBS and addition of fresh media. At 5–7 days post-infection, cells were freeze-thawed and the resulting cell culture supernatant (BP1) was used to reinfect fresh cells. Such blind passages continued until appearance of CPE. (c) Detection of PPRV and FMDV-specific genome in mock-infected and virus-infected cell culture supernatants at BP6.

As shown in **Fig 1A**, compared to the negative control (Minimum Essential Medium, MEM), a single clinical specimen was found to be positive for both PPRV (337 nt) and FMDV (327 nt)-specific gene segments which confirmed PPRV/FMDV mixed infection in the affected goatherd. One animal was found negative for PPRV but not FMDV genome, suggesting it might have been vaccinated against PPR.

BHK-21 and Vero cell lines are the established cell lines, commonly used for isolation of FMDV and PPRV respectively. For virus isolation, primarily, the specimens were propagated separately in BHK-21 and Vero cells but no virus-specific cytopathic effect (CPE) could be observed even after eight blind passages (BP). Later, one of the clinical specimens that was positive for both FMDV and PPRV-specific gene segments was serially propagated in cocultured Vero/BHK-21 cells. At BP6, CPE characterized by cell rounding, ballooning and degeneration was evident only in the virus-infected but not in mock-infected cells (**Fig 1B**) that suggested replication/adaptation of the virus(es) in the cocultured Vero/BHK-21 cells. Similar to the clinical specimens, virus-infected but not mock-infected cell culture supernatant (BP6) was also found to be positive for both PPRV and FMDV-specific gene segments (**Fig 1C**). Since the cells were extensively washed with phosphate buffered saline (PBS) following each infection, carrying virus input up to BP6 is unlikely, therefore, detection of both the viral genomes at this stage (BP6) represented evidence of adaptation of both PPRV and FMDV in the cell culture system.

### Purification of PPRV

**Fig 2:**
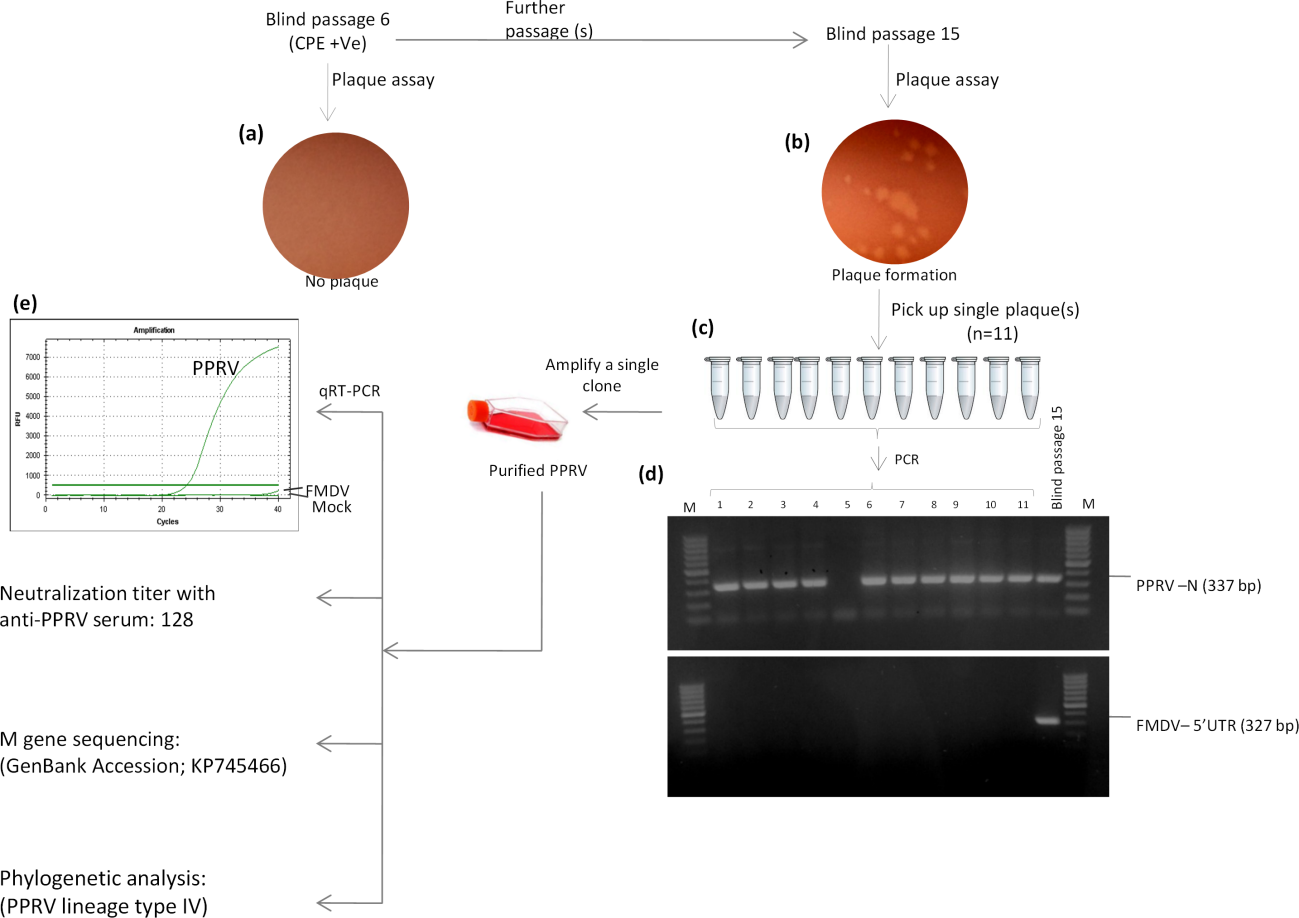
PPRV purification and characterization. (a) Plaque assay at lower passage (BP6). (b) Plaque assay at higher passage (BP15). (c) Plaque purification: eleven different plaques were picked and resuspended in 500 μl MEM. (d) Amplification of PPRV (upper panel) and FMDV (lower panel)-specific gene segments from individual plaques by PCR. (e) qRT-PCR for detection of PPRV and FMDV-specific genomes in plaque purified PPRV (purity check).

Rather than at lower passage (BP6) (**Fig 2A**), infected cell culture supernatant at higher passage (BP15) (**Fig 2B**) formed plaques in Vero cells. We picked up 11 plaques **(Fig 2C)** and tested them for PPRV and FMDV-specific gene segment by PCR where all the plaques (except plaque 5 where no amplification was observed) belonged to PPRV **(Fig 2D,** upper panel). No FMDV-specific amplification could be observed in any plaque (**Fig 2D,** lower panel). Plaque number 5 which was negative for both PPRV and FMDV genomes, also did not produce CPE, probably it was devoid of any virus particle due to improper picking. To prepare a stock of the purified PPRV, one of the plaques was further amplified in Vero cells and stored in -80°C deep freezer until use.

### Characterization of purified PPRV

Purified PPRV was neutralized with known anti-PPRV serum with a neutralization titer of 128 suggesting viral homology with the Indian vaccine strain. When tested by quantitative real-time PCR (qRT-PCR), purified PPRV stock was found to be free from any FMDV-specific genome **(Fig 2E),** suggesting successful purification of PPRV. Further, the matrix (M) protein gene of PPRV was amplified by PCR and directly sequenced. High quality sequences were deposited in GenBank under Accession Number KP745466. On BLAST search, the sequences showed highest homology (99.66%) with PPRV/India/TN/Gingee/2014 (GenBank Accession, KR261605.1) and phylogenetically grouped under lineage type IV PPRV. Purified PPRV was deposited at National Centre for Veterinary Type culture Collections (NC-VTCC), Hisar, India, under Accession Number VTCC AVA 154.

### Purification of FMDV

We employed several strategies (**Table 1**) to eliminate PPRV from the virus mixture and hence to obtain purified FMDV. Though FMDV genome was consistently detected from BP1 to BP15, no FMDV-specific plaques could be observed both in BHK-21 and Vero cells with all the established methods of FMDV plaque assays employed [28–30].

**Table 1:** Strategies employed for purification of PPRV and FMDV from the virus mixture.

S. N.	Strategy	Remark
1.	Treatment with organic solvents to eliminate enveloped virus (PPRV)	Unsuccessful (because the concentration of the organic solvents required for complete inactivation of the virus particles was toxic to the target cells
2.	Removal of hemagglutinating virus (PPRV)	Unsuccessful in completely adsorbing PPRV
3.	Plaque assay to purify FMDV and/or PPRV	Successful for PPRV but no FMDV-specific plaques could be recovered both in Vero and BHK21 cells
4.	Limiting dilution assay to purify PPRV and FMDV	Unsuccessful
5.	Neutralization with anti-PPRV serum to eliminate PPRV	Limited success. At higher passage, when FMDV starts forming CPE, defective interfering particles may appear that interfere with plaque formation as well as facilitating extinction of standard FMDV genome.
6.	Serial blind passage of the virus mixture to eliminate one of the coinfecting viruses	Not quite successful, partly depends on cell type used
7.	Viral RNA transfection (virus mixture) into target cells	Most efficient methods for eliminating a negative stranded RNA virus from the virus mixture and hence purification of a positive stranded RNA virus.

PPRV is an enveloped virus and considered to be sensitive to the organic solvents. Therefore, in order to purify FMDV from the virus mixture, we first attempted to inactivate the PPRV with ethanol and isopropanol (80% ethanol, 5% isopropanol) [31] and then infected to BHK-21 and/or Vero cells for amplification. At a noncytotoxic concentration (<1.25%), the ethanol and isopropanol treatment was ineffective in completely inactivating the PPRV replication as both PPRV and FMDV genome were detectable in infected cell culture supernatant (data not shown). Other methods of purifying FMDV such as hemagglutination with chicken red blood cells (RBCs) (to eliminate PPRV) and limiting dilution assay were also not quite successful (**Table 1**).

**Fig 3:**
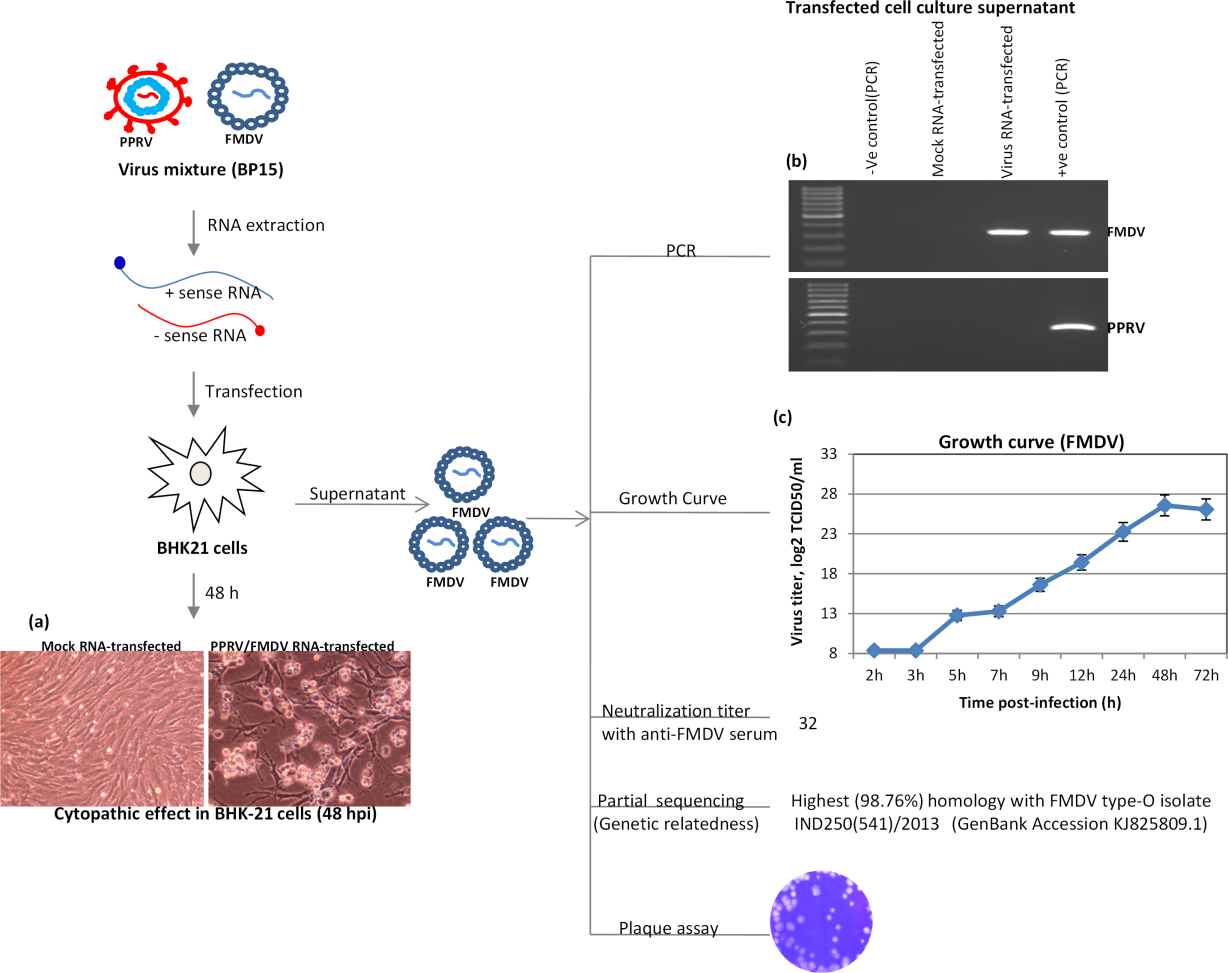
FMDV purification and characterization. RNA was extracted from 400 μl of the cell culture supernatant collected from either the mock-infected or BP15 virus-infected cells, followed by transfection in BHK-21 cells. (a) Cytopathic effect in mock-RNA-transfected and virus RNA-transfected BHK-21 cells at 48 hpt. (b) Amplification of FMDV-specific (upper panel) but not PPRV-specific (lower panel) gene segments by PCR in transfected cell culture supernatants. GMEM and virus mixture (BP15) were used respectively as negative and positive controls for RNA isolation and PCR. (c) BHK-21 cells were infected, in triplicates, at MOI of 1 for 1 h followed by washing and addition of fresh GMEM. The infectious progeny virus particles released in the infected cell culture supernatants at indicated time points were quantified by determining TCID_50_ in BHK-21 cells. (d) Plaque formation by purified FMDV.

FMDV is a positive stranded RNA virus, therefore it is believed to produce infectious FMDV upon transfection of its RNA into the target cells [32]. Upon RNA (virus mixture) transfection to BHK-21 cells, we observed CPE only in the cells that received RNA from the virus mixture but not in the cells that received mock-RNA (**Fig 3A**). At 48 h-post-transfection (hpt), when the transfected cell culture supernatant was evaluated for FMDV and PPRV-specific gene segments, only FMDV, but not PPRV, could be detected (**Fig 3B**) suggesting release of only FMDV virions from the transfected cells and hence elimination of the PPRV.

### Characterization of purified FMDV

When using FMDV serotype-specific PCR primers, amplification was observed only with type O-specific primers (data not shown), therefore the virus belonged to the FMDV serotype “O”. The PCR product was subsequently gel purified and directly sequenced using reverse PCR primer. The sequences were deposited in the GenBank under Accession Number KP745467, which showed highest homology (98.76%) with FMDV type-O isolate IND298(634)/2013 (GenBank Accession KJ825804.1). The small 5’ untranslated region (UTR) which was amplified using FMDV serotype independent primers was also sequenced using reverse PCR primer and accessioned with the GenBank Accession Number KP757899. The partial 5’UTR sequences also showed highest similarity (100%) with FMDV type-O isolate IND250(541)/2013 (GenBank Accession KJ825809.1).

To determine the growth curve, BHK-21 cells were infected with FMDV at multiplicity of infection (MOI) of 1 and the infectious FMDV released in the cell culture supernatant at various time points was titrated by determining the tissue culture infective dose 50 (TCID_50_). As shown in **Fig 3C**, there was no significant increase in viral titers in the supernatants that were collected at 2 h post-infection (hpi) and 3 hpi. The detectable amount of virus particles at these earlier time points (2 hpi and 3 hpi) represented the background level (input) which remained attached with vessel/cells even after washing following infection. However, there was a sharp rise in viral titer in supernatants at 5 hpi or later which indicated that new progeny virus particles started releasing from the infected cells somewhere around 5 hpi as one full cycle of the viral replication had completed (**Fig 3C**). FMDV life cycle is ~ 5 h [33–36], therefore these findings are in agreement with the previous reports.

Purified FMDV produced plaques within 48 h in BHK-21 cells **(Fig 3D)** and was neutralized with known anti-FMDV serum at a titer of 32. These results reflected that there was no significant antigenic and genetic diversity in the FMDV under study. Purified FMDV was deposited to NC-VTCC under Accession Number VTCC AVA 153.

### Long-term copersistence of FMDV/PPRV in cocultured cells

The FMDV and PPRV specific genomes could be detected even at much higher passage (BP15) (**Fig 2D),** which again suggested that cocultured Vero/BHK-21 cells were able to support long-term copersistence of FMDV and PPRV.

**Fig 4:**
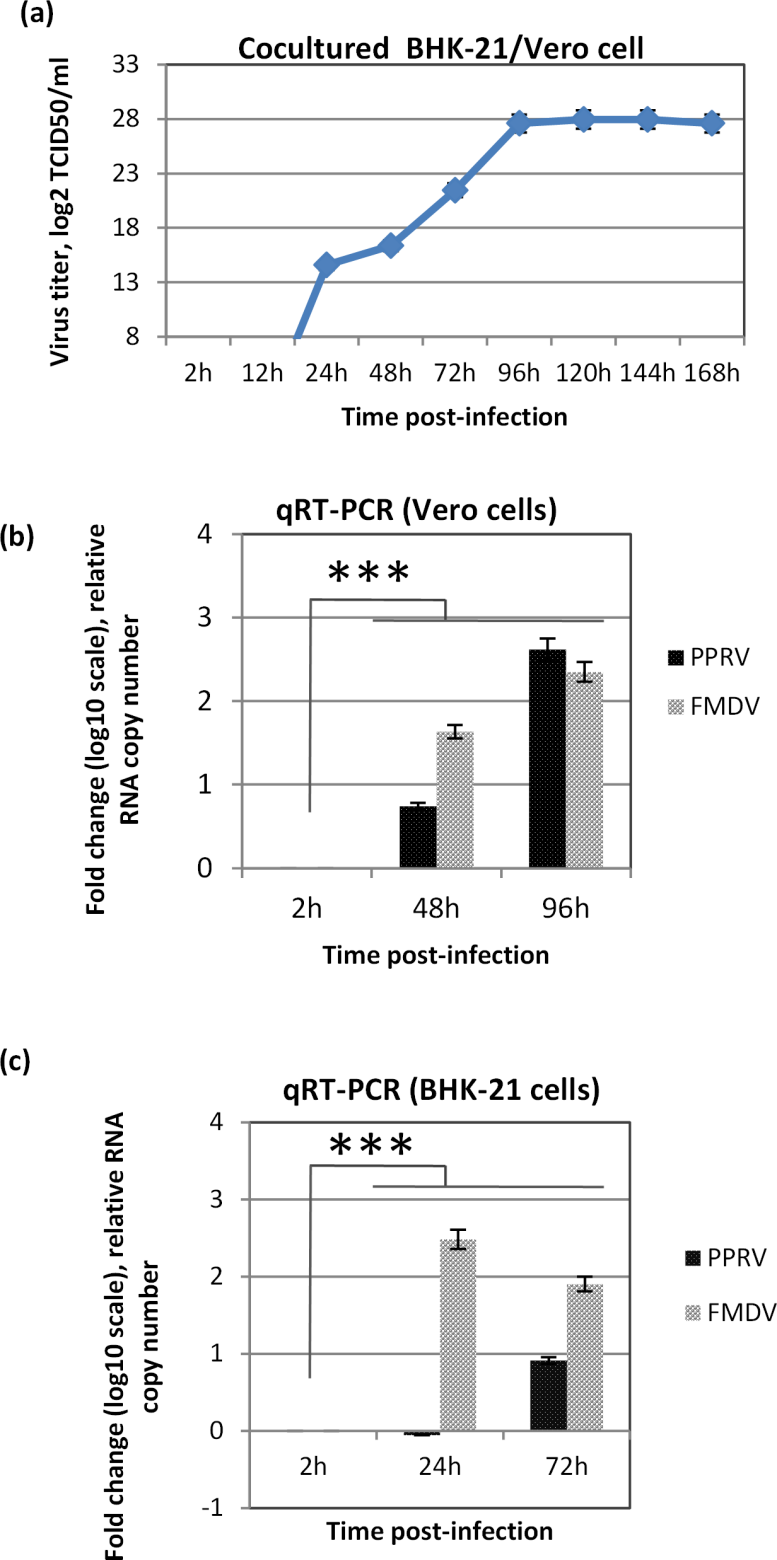
Copersistence of FMDV/PPRV in cocultured BHK-21/Vero cells. Confluent monolayers of cocultured BHK-21/Vero cells or single cell types (Vero or BHK-21 cells) were infected, in triplicates (12 well cell culture plate), with 100 μl of the cell culture supernatant (BP15) for 2 h followed by washing with PBS and addition of fresh media. Virus release in the infected cell culture supernatant (cocultured BHK/Vero cells) at indicated time points was quantified by determining TCID_50_ (a). Infected cell culture supernatants from Vero (b) and BHK-21 (c) cells at indicated time points were tested for PPRV and FMDV-specific genomes by qRT-PCR. The viral RNA levels, expressed as threshold cycle (CT) values, were analyzed to determine relative fold change in RNA copy number over 2 hpi. Error bars indicate SD. Statistical analysis was conducted with Student’s *t* test (*** = P<0.001).

As compared with 2 hpi and 12 hpi, sharp rise in the virus titer at 24 hpi in the infected cell culture (cocultured cells) supernatant suggested the formation and release of new progeny virus particles at this stage (**Fig 4A).** The virus titer increased until 96 hpi, it became stable between 120 hpi and 144 hpi and then started to be gradually decreased at 168 hpi.

In order to evaluate whether the virus(es) adapted to cocultured Vero/BHK-21 cells were able to replicate in a single cell type, BHK-21 and Vero cells were separately infected with the virus mixture (BP15) and the virus released in the infected cell culture supernatant at various time points was quantified by qRT-PCR. As compared to 2 hpi, a highly significant increase in the relative RNA copy number of both PPRV and FMDV were observed at later time points in both Vero (At 96 h, 414 and 223 fold increase respectively in PPRV and FMDV) **(Fig 4B)** and BHK-21 (303 fold increase in FMDV at 24 h and 8 fold increase in PPRV at 72 h) (**Fig 4C**) cells suggesting that both the viruses were able to replicate in a single cell type.

### Viral interference

**Fig 5:**
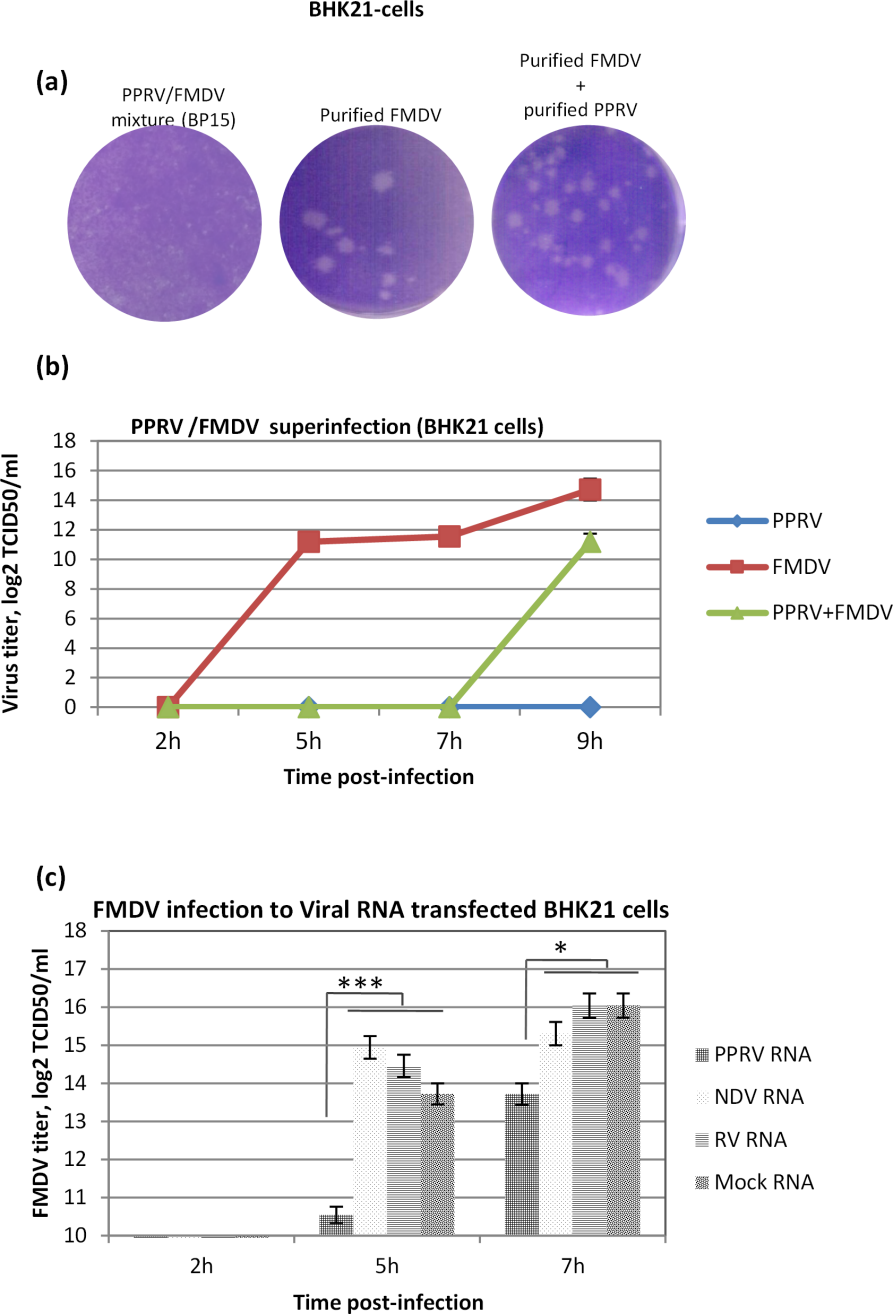
PPRV interferes FMDV replication. (a) FMDV plaque assay in BHK21 cells (b) Confluent monolayers of BHK-21 cells were infected, in triplicates, with PPRV at MOI of 1. At 12 hpi, the cells were superinfected with FMDV at MOI of 1 for 1 h followed by washing with PBS and addition of fresh media. The infectious progeny virus particles released in the supernatant at indicated time points were quantified by determining TCID_50_ in BHK-21 cells. (b) BHK-21 cells were transfected, in triplicates, with PPRV, NDV, RV or mock (cellular) RNA. At 6 hpt, cells were washed with PBS and infected with FMDV at MOI of 1 followed by washing with PBS and addition of fresh media. Infectious progeny virus particles released in the infected cell culture supernatants were quantified by determining TCID_50_. Results shown are the averages from at least three independent experiments. Error bars indicate SD. Statistical analysis was conducted with Student’s *t* test (*** = P<0.001, * = P<0.05).

Purified FMDV, but not virus mixture (BP6 to BP15) formed plaques in BHK-21 cells (**Figs 3D and 5A**), suggesting that the PPRV would have interfered with FMDV plaque formation. However, purified PPRV, when coinfected with purified FMDV, did not interfere FMDV plaque formation **(Fig 5A),** suggestive of other possibilities such as generation of defective interfering (DI) virus particles.

In order to demonstrate viral interference between PPRV and FMDV, BHK-21 cells were either mock-infected or infected with PPRV and then subsequently superinfected with FMDV and the virus released in the supernatant was quantified. As shown in **Fig 5B**, as compared to PPRV/FMDV superinfection, FMDV alone replicated much faster and at higher titer suggesting PPRV interfered with FMDV replication. It’s worth mentioning here that in BHK-21 cells, as compared to PPRV which takes ~ 72 h (data not shown) in completing its one cycle, FMDV life cycle is very short (~ 5 h), therefore, the infectious virus released in the coinfected cell culture supernatant was believed to be FMDV only.

To further explain the mechanism of viral interference, BHK-21 cells were transfected with PPRV (single stranded negative sense RNA), NDV (single stranded negative sense RNA from same family) or rotavirus (RV) (double stranded RNA) RNA and then infected with FMDV. As shown in **Fig 5C**, prior transfection of PPRV RNA, but not NDV and RV RNA, significantly inhibited FMDV replication in BHK-21 cells suggesting that the PPRV induced interference was specifically directed against FMDV.

**Fig 6:**
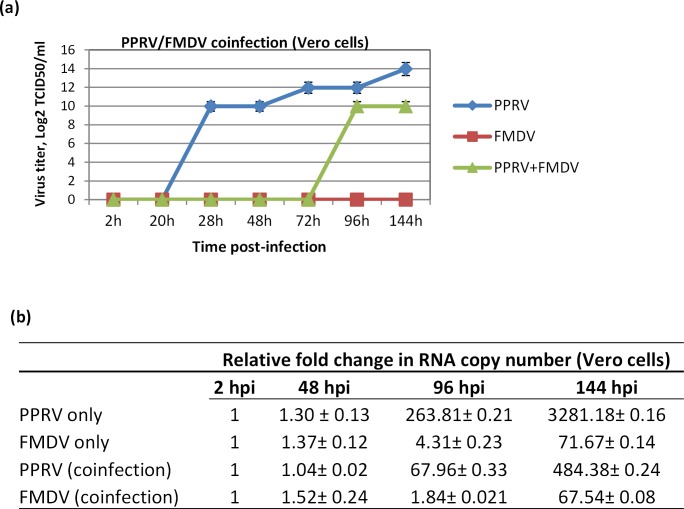
FMDV interferes with PPRV replication. Confluent monolayers of Vero cells were infected, in triplicates, with a single virus (PPRV or FMDV) or coinfected with PPRV/FMDV, each at MOI of 1 for 2 h followed by washing with PBS and addition of fresh media. Virus released in the supernatants at indicated time points were quantified by (a) determination of TCID_50_. (b) Relative fold change in RNA copy number (over 2 hpi).

The viral interference phenomenon was also examined vice-versa that is if FMDV interfered with PPRV replication. Vero cells, which are highly permissive for PPRV and in which FMDV replicates very poorly, were coinfected with PPRV/FMDV and the virus released in the supernatant was quantified. As shown in **Fig 6A**, as compared to coinfected cells, there was significantly higher virus titer in the cells that were infected with PPRV only, suggesting that the FMDV inhibited PPRV replication. Similarly, when examined by qRT-PCR, the supernatant from PPRV alone infected cells showed significantly higher increase in RNA copy number as compared to PPRV/FMDV coinfection (~ 260-fold versus ~ 67-fold at 96 hpi and ~ 3281 fold versus ~ 484 fold at 144 hpi) (**Fig 6B**).

Interestingly, purified FMDV did not produce any CPE in Vero cells and hence there was no measurable virus when titrated by determining TCID_50_ (Vero cells) (**Fig 6A**). However, when the supernatants were examined for FMDV genome by qRT-PCR, as compared to 2 hpi where no progeny virus particle was believed to be produced, a significant increase in FMDV copy number was observed at 96 hpi (~ 4 fold) and at 144 hpi (~71 fold) (**Fig 6B**) suggesting that the FMDV has replicated and produced new progeny virus particles, though they are noncytolytic (**Fig 6A**).

### Virus persistence, exclusion and accommodation

**Fig 7:**
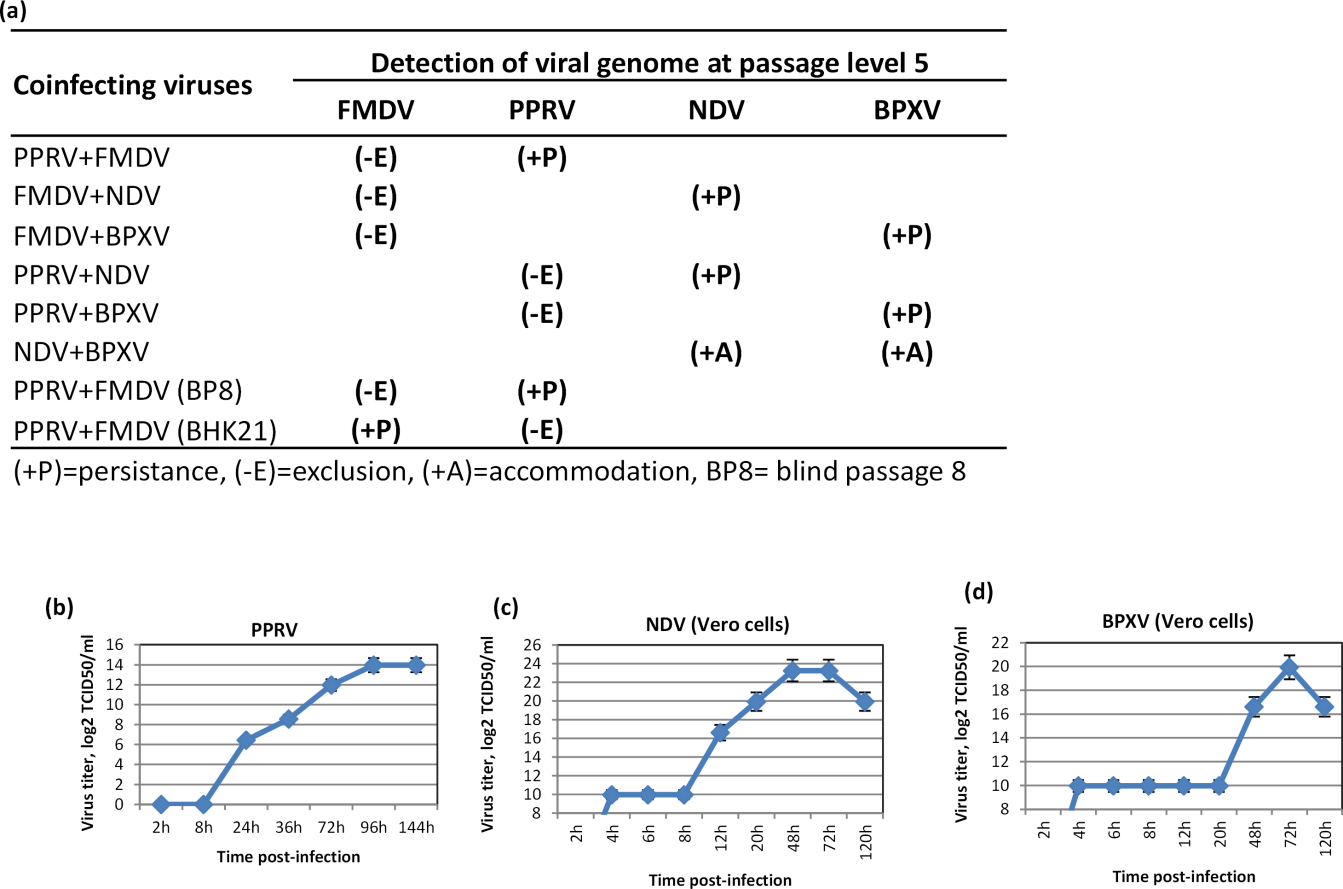
Fate of long-term *in vitro* viral coinfections. (a) Vero cells were infected with indicated combinations of the viruses at MOI of 1. Infected cell culture supernatant was harvested at 7–8 days post-infection or earlier if >50% CPE was observed and 200 μl of it was used for next passage. Five such sequential passages were made. The infected cell culture supernatant from 5^th^ passage was subjected for amplification of respective viral genome by PCR. (b) **Growth curve of PPRV.** Confluent monolayers of Vero cells were infected, in triplicates, with PPRV at MOI of 1 for 2 h followed by washing with PBS and addition of fresh media. The infectious progeny virus particles released in the supernatant at indicated time points were quantified by determination of TCID_50_ in Vero cells. (c) **Growth curve of NDV.** Confluent monolayers of Vero cells were infected, in triplicates, with NDV for 1 h at MOI of 1 followed by washing with PBS and addition of fresh media. The infectious progeny virus particles released in the supernatant at indicated time points were quantified by determining TCID_50_ in Vero cells. (d) **Growth curve of BPXV.** Confluent monolayers of Vero cells were infected, in triplicates, with BPXV at MOI of 1 for 1 h followed by washing with PBS and addition of fresh media. The infectious progeny virus particles released in the supernatant at indicated time points were quantified by determining TCID_50_ in Vero cells.

After explaining the viral interference phenomenon, we were interested in examining the fate of coinfected viruses on long-term *in vitro* passage. We therefore performed coinfection experiment with several combinations of the viruses **(Fig 7A)**, all of which are believed to cause an acute infection in natural host. When tested for viral genome at passage level 5, in most combinations, only one coinfected virus survived (persisted) whereas the other was eliminated (exclusion), with the only exception of Newcastle disease virus (NDV) and buffalopox virus (BPXV) coinfection where both the viruses persisted (accommodation) **(Fig 7A)**.

In order to understand virus persistence, exclusion and accommodation more precisely, we also performed growth curve of the individual virus, where, following infection, the virus released in the supernatant at different time points was quantified by determining TCID_50_. Though virus life cycle may depend on nature of the virus, MOI of virus and cell types used, at similar MOI, new progeny virus particles (life cycle) were detected in the infected cell culture supernatant at ~5 hpi in FMDV **(Fig 3C)**, ~24 hpi in PPRV **(Fig 7B)**, ~12 hpi in NDV **(Fig 7C)** and ~48 hpi in BPXV **(Fig 7D).** Since the life cycle of different viruses vary, the fast replication of one virus would have exhausted the host cell resources (cell death) and hence exclusion of second coinfecting virus would have occurred.

### Extinction of standard FMDV genome on sequential high passage

**Fig 8:**
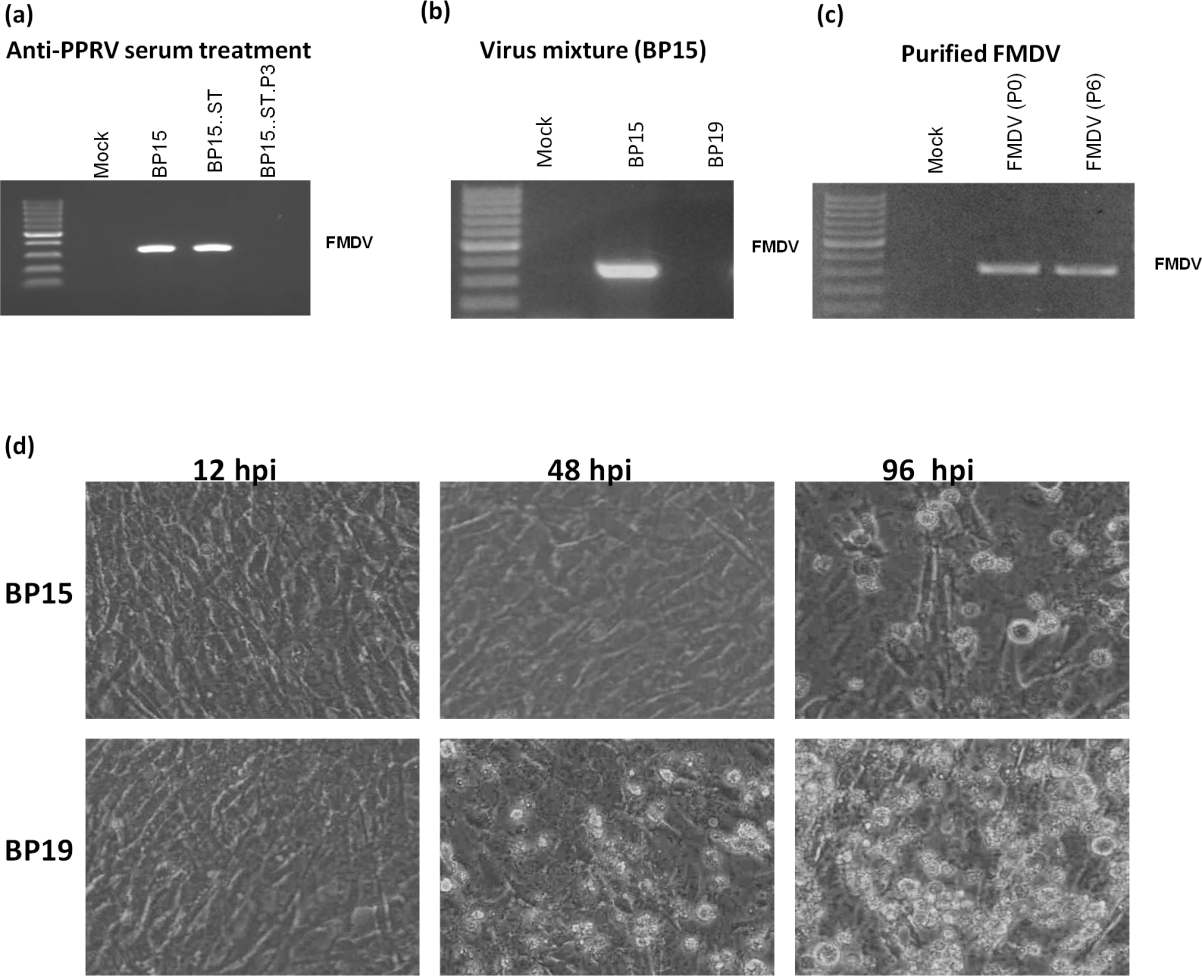
Extinction of standard FMDV genome at higher passage. (a) Detection of FMDV genome in mock-infected, BP15-infected, BP15.ST (anti-PPRV serum treated BP15)-infected or BP15.ST.P3 (Sequential 3 time passage of BP15.ST)-infected cell culture supernatant. (b) Detection of FMDV genome in mock-infected, BP15-infected and BP19-infected cell culture supernatant. (c) Detection of FMDV genome in mock-infected, purified FMDV(P0)-infected and six time passaged FMDV (P6)-infected cell culture supernatant.

As indicated above, we also performed anti-PPRV serum treatment for purification of the FMDV from the virus mixture. The virus mixture (BP15) was treated with anti-PPRV serum and the resulting mixture was used to infect BHK-21 cells. At 4–5 days post-infection, when there was a clear CPE, the infected cell culture supernatant was harvested by freeze-thaw and named BP15.ST. Like BP15, BP15.ST also did not form plaques (data not shown) but surprisingly when further passaged (P = 3), FMDV genome was undetectable by RT-PCR (5’UTR-based PCR primers) (**Fig 8A**). Likewise, when the original virus mixture (BP15) was further passaged, the FMDV genome was also undetectable at passage level 4 (BP19) (**Fig 8B**) indicating this phenomenon was simply due to higher passage rather than anti-PPRV serum treatment, probably occurred by generation of DI particles (**Table 2**). It was also observed that, as compared to virus at BP15 which produced CPE at ~ 96 hpi, virus at BP19 produced rapid CPE (within 48 hpi) (**Fig 8C**). Interestingly, the FMDV that was purified from the virus mixture by RNA transfection, even when further passaged (P = 6), behaved normally as it formed plaques and FMDV genome was still detectable, like those observed with wild type virus (**Fig 8C**). The data indicated the possibilities of generation of DI particles at higher passage which interfered with FMDV plaque formation. It could also be concluded that as compared to antibody neutralization, the RNA transfection method is more efficient in purifying FMDV from PPRV/FMDV mixture (**Table 2).**

**Table 2:** FMDV purification from the virus mixture: Comparison of RNA transfection and anti-PPRV serum treatment.

Purification method	Source material	Cytopathic effect	FMDV genome	Extinction of standard FMDV genome on subsequent high passage*	Plaque formation in BHK21 cells
**RNA transfection**	CS	(-)	(+)	NA	(-)
	BP8	(-)	(+)	NA	(-)
	BP15	(+)	(+)	**(-)**	**(+)**
**Anti-PPRV serum treatment**	CS	(-)	(+)	NA	(-)
	BP8	(-)	(+)	NA	(-)
	BP15	(+)	(+)	**(+)**	**(-)**

CS = clinical specimens, BP8 = Blind passage 8, BP15 = Blind passage15, (-) = negative, (+) = positive, NA = not applicable.

* Formation of DI particles.

### Revisiting the FMDV purification directly from the clinical specimens

We further examined whether FMDV isolation/purification is possible directly from the clinical specimens and from virus at BP8 (lower passage). As shown in **Table 2**, virus mixture from the original clinical specimen as well as at BP8, either treated with anti-PPRV serum or subjected to transfection, did not produce CPE in BHK-21 cells, suggesting FMDV remained noncytolytic at lower passage and that the CPE observed at lower passage level was primarily due to PPRV. Since FMDV genome was detected at each passage, the virus replication was expected to occur consistently.

## Discussion

Mixed viral infections may influence disease severity, transmissibility, immunopathology and vaccine effectiveness [37]. Both PPR and FMD are economically devastating diseases with similar epidemiology. Our results are the first report that illustrated a PPRV/FMDV mixed infection in the field goats. We observed about 50% mortality in the affected goatherd. The mortality rate in PPR affected goatherds varies from 10–90% [38], whereas FMDV infection does not causes any mortality except some young animals which may die due to myocarditis [39], therefore contribution of FMDV in increasing or decreasing the disease severity could not be ascertained. Except few exceptions [40], where noted, previous reports also did not indicate any increased disease severity that might be attributed to the mixed viral infections [41–44]. Besides influencing disease severity, mixed infection may also influence rate of virus replication, virus distribution inside various tissue/cell types and, virus dissemination and transmission [20]. In contrast to acute infections where virus is eventually eliminated through immune response or destruction of host, virus survives for long time in persistently infected animals and may transmit to other animals in contact with. Movement of livestock and trade plays a key role in the spread of FMD [45,46]. Because of inapparent nature of the disease, small ruminants are usually been ignored for their epidemiological role in transmission of FMD [16,47]. However, FMDV-infected animals may become carriers and therefore represent a risk of entry of FMD to disease-free countries [48]. Overlapping epidemiology of FMD and PPR [14] may further complicate the matters as PPRV [49], and particularly FMDV [50] infected animals may develop persistent infection.

The sensitivity of the virus isolation varies depending of cell type and a single cell culture is less useful for the isolation of multiple viruses [3]. Different cell types grown together in a single monolayer (cocultured cells) have been advocated for detection (immunofluorescence) of multiple viruses [51,52], though their precise role in virus isolation is not well documented. Our attempt isolating PPRV and FMDV from the mixed infection in goats using a single cell type (BHK-21 or Vero) was unsuccessful. Cocultured Vero/BHK-21 cells in which both PPRV and FMDV got adapted, represents an efficient system for simultaneous isolation of PPRV and FMDV from the mixed infections. Adaptation of a natural virus isolate to the cell culture system entails a modification of the genetic composition of the viral population [53]. Whether it’s just a stochastic event or some specific interactions which made PPRV/FMDV adaptation into cocultured cells but not in single cell type need further investigations.

Due to overgrowth of one of the coinfecting virus, successful purification of multiple viruses from the coinfected cases remains a challenge. This may be partly overcome by plaque purification and antibody neutralization [54], though it’s not always successful, as virus usually remains noncytolytic at lower passage levels. Since the virus mixture did not produce any FMDV-specific plaques both in Vero and BHK-21 cells at any passage level, we employed some other possible techniques for FMDV purification viz; treatment with organic solvents to eliminate PPRV (an enveloped virus) [31], hemagglutination to remove PPRV, endpoint dilution assay to isolate PPRV and/or FMDV, neutralization with anti-PPRV serum to eliminate PPRV and, finally, transfecting viral RNA mixture (BP15) to BHK-21 cells where only positive stranded RNA (FMDV) but not negative stranded RNA (PPRV) was expected to form infection virions [32,55]. As indicated in **Table 1**, only antibody neutralization and RNA trasfection techniques were successful in eliminating PPRV from the virus mixture, though the virus purified from first method did not form plaques. Among the mixture of a negative and a positive stranded RNA virus, RNA transfection, which we have employed for the first time in this study, appears to be the most efficient method in eliminating the negative stranded RNA virus (PPRV) and hence purification of the positive stranded RNA virus (FMDV). It could possibly provide insights in the isolation and purification other type of viruses from natural mixed infections.

RNA viruses produce DI particles at high MOI serial passage in cell culture [56]. These are spontaneously generated virus mutants which have defective/deleted genomes [57], replicates faster than the standard virus [58], and usually require another fully functional virus particle (the helper virus) to coinfect a cell with it for effective replication [59]. DI particles have also been described during high sequential passage of FMDV [60]. Moreover, even in the absence of any standard viral RNA, complementation between two defective RNA genomes has been shown to cause cytopathology [58]. Therefore we believed that the absence of standard FMDV genome (but rapid CPE) at higher passage (≥BP 19) in our study was due to DI particle formation. Deletions in most of the picornaviruses DI genomes are located in the 5’ portion of viral RNA [61,62]. On sequential passage with DI particles, the levels of standard RNA progressively declines and may not be detectable by RT-PCR [53,58,63] which we have seen in our study at ≥BP19.

Plaque formation by a standard RNA virus can be inhibited by DI particles [64]. Rather than high passaged virus mixture, purified FMDV (by RNA transfection) alone, or when coinfected with purified PPRV, formed plaques in BHK21 cells suggested that it was not PPRV but probably DI particles that interfered with FMDV plaque formation in the virus mixture. Since FMDV genome was detectable in the purified FMDV even at >6 passage, with the mechanisms unknown, it may be concluded that the transfection method acts as a filter to remove the entities (probably DI particles) that interfere with plaque formation. As PPRV plaques could be seen at any passage level even at >BP15, it was also concluded that DI particles did not interfere with PPRV plaque formation. Further studies on morphological, antigenic and genetic characterization of DI particles are required which are beyond the scope of this manuscript.

We further examined whether FMDV isolation/purification is possible directly from the clinical specimens. Unlike BP15 virus, original clinical specimen as well as virus at BP8 (lower passage), either treated with anti-PPRV serum or subjected to transfection, did not produce CPE in BHK-21/Vero cells, suggesting FMDV remains noncytolytic at lower passage level (BP = 8) and that the CPE observed at lower passage level was primarily due to PPRV. Since FMDV genome was consistently detected up to BP15, a productive FMDV replication was expected to occur consistently during each passage in the cell culture. Thus it was concluded that both transfection and anti-PPRV serum treatment are unlikely to produce cytolytic FMDV at lower passage level. Contrary, in a previous report, authors were able to rescue cytolytic FMDV by directly transfecting clinical specimens-derived viral RNA into primary bovine thyroid (BTY) cells [32], though in certain cases, where quantity of viral RNA was less, further blind passages were required until CPE was seen in BTY cells [32]. Primary BTY cells are considered highly sensitive for FMDV isolation [65] and therefore, the difference in our and previous report might be due to the intrinsic ability of the BTY cells to support FMDV isolation.

FMDV under study showed highest homology (98.76%) with FMDV type-O isolate IND250(541)/2013 and it was neutralized with known anti-FMDV (FMDV type O, A and Asia-1 vaccinated serum) serum at a titer of 32. Likewise, PPRV showed highest homology (99.66%) with PPRV/India/TN/Gingee/2014 (phylogenetically grouped under lineage type IV PPRV prevalent in India) and neutralized with known anti-PPRV serum at a titer of 128 suggesting coinfection did not induce any significant genetic or antigenic diversity.

Viral interference, a phenomenon for which a cell infected by a virus becomes resistant toward a second superinfecting virus, has been shown to exists between viruses that may or may not be closely associated [1,20,66–69]. Several factors such as DI particles, small interfering RNA (siRNA), competition for cellular factors and innate immune response may implicate in viral interference [20]. Interferons produced from one infected cell may migrate to other nearby cells and induce antiviral-resistant state in it. In most instances of concurrent infections, the mechanism of dominance of one virus is mediated via interferon (host-mediated) [70,71], however, direct interference (virus component-mediated) may also occur [22]. Our *in vitro* coinfection experiments with PPRV/FMDV in Vero cells indicated that FMDV interferes PPRV replication. In coinfected Vero cells, FMDV replicated (enhanced RNA copy number over times) but did not produce any cytolytic FMDV. Nevertheless, noncytolytic phenotype of FMDV has been reported following high sequential passages in BHK-21 cells [72]. Noncytolyitc virus phenotype in non-permissive cell lines is not uncommon [73], however, how FMDV produces noncytolytic phenotype in Vero cells warrants further investigation.

Viral interference was also examined vice versa that is if PPRV interferes FMDV replication. Rather than coinfection, superinfection of FMDV in PPRV-infected BHK-21 cells resulted in viral interference. PPRV replicates very poorly in BHK-21 cells where the evidence of progeny virus particles production cannot be observed before 72 hpi and the CPE cannot be observed until 144–168 hpi. Contrarily, FMDV replicates very fast in BHK-21 cells (life cycle ~ 5h) and produce CPE within 8–10 hpi. Extremely slow replication of PPRV in BHK-21 cells probably did not accumulate significant nucleic acid (PPRV RNA) inside infected cells and hence no viral interference was observed. However, superinfection (where PPRV infection was performed 12 h prior to FMDV infection), allowed sufficient time for accumulation of PPRV gene products and hence viral interference to occur. To further explain the mechanisms of viral interference, rather than virus infection, we performed nucleic acid (RNA) transfection. Prior transfection of PPRV but not NDV and RV RNA into BHK-21 cells resulted in reduced FMDV replication suggesting that the FMDV replication was specifically interfered by PPRV RNA and that the interference is predominantly mediated through the virus instead of cellular factors. Taken together data suggested that the *in vitro* viral interference depends on the nature of the virus, cell types used for coinfection and time interval of primary and secondary virus infection [20,74,75].

Despite viral interference observed in single cell types, we observed long-term co-persistence of PPRV/FMDV in cocultured cells. FMDV [36] and PPRV [14] both can efficiently infect epithelial cells and lymphocytes, though other cell types may also get infected [36]. It means *in vivo*, where wide varieties of cell types are available, the long-term copersistence of FMDV and PPRV may occur. Viral interference is associated with but not exclusive to persistent infection [20]. Cocirculation of PPRV and FMDV in the same geographical area increases the opportunity for interviral interactions and hence heterologous viral interference to take place. A somewhat similar observation has been observed following superinfection of chikungunea virus (CHIKV) to dengue virus (DENV) infected mosquitoes where both the viruses were shown to persist together [76] in presence of heterologous viral interference [77]. These observations indicate that these viruses share similar cellular mechanisms that control viral and cellular factors required for efficient virus replication. Such cellular mechanisms are associated with host factors that regulate but do not eliminate virus infection, such as those involved in innate antiviral defense and RNAi [20]. Prior infection with rhinovirus in humans has been shown to affect influenza virus epidemiologly [1]. To address the significance of PPRV/FMDV mixed infection in modifying transmission and epidemiology of the disease in natural population, further studies on long-term *in vivo* persistence of these two acute pathogenic viruses are required. Nevertheless, our mixed virus infection study on these two economically important animal viruses will contribute in establishing guidelines for trading of livestock that could further transmit the infections and epidemics in disease free countries.

No significant data are available on the fate of *in vitro* coinfection on long-term passage. Based on our viral interference experiments, we hypothesized that only one virus should persist on long-term *in vitro* passage. When tested for viral genome at passage level 5, in most combinations, only one coinfecting virus persisted and another was excluded, with the only exception of NDV/BPXV coinfection where both the viruses persisted (accommodation). Purified FMDV, though replicated (increase viral RNA copy number following infection), did not produce CPE (nonytolytic) in Vero cells, therefore its exclusion was likely to occur in NDV/FMDV, PPRV/FMDV and BPXV/FMDV coinfections. In PPRV/NDV coinfection, because of relatively long life cycle of PPRV, its exclusion occurred as anticipated. In BPXV/PPRV coinfection, though PPRV replicated much faster (life cycle <24 h) as compared to BPXV (life cycle ~ 48h), but contrarily BPXV persisted and PPRV was excluded. PPRV though started producing infectious virions in <24 hpi, did not produce observable CPE until 96 hpi, whereas BPXV produced CPE almost at the same time (48 hpi) when evidence of new infectious progeny virus particles was seen in the infected cell culture supernatant. Due to faster development of the CPE, BPXV did not allow sufficient replication of PPRV (due to competition of cell resources) and hence PPRV was excluded. Role of DI particles and heterologous viral interference in excluding one coinfecting virus appears to be a complex intracellular mechanism and need further investigations. Interestingly, despite huge difference in duration of the life cycle, both NDV and BPXV persisted (accommodated) reflecting requirement of different cellular factors to complete the viral replicative cycles. Taken together, it may be concluded that besides nature of the virus and cell types, *in vitro* viral copersistence and exclusion depends on the dynamics of virus replication and CPE formation. Whether NDV/BPXV can accommodate each other on further high passages as well as mechanism underlying the accommodation needs to be investigated which is beyond the scope of this manuscript.

Unlike cocultured cells, subsequent passage of BP8 virus (PPRV+FMDV) in a single cell type (Vero) resulted in FMDV exclusion. Further, when we performed long-term (P = 5) coinfection (purified PPRV/ purified FMDV) experiments in BHK-21 cells, PPRV but not FMDV excluded (data not shown). Therefore, first passaging in cocultured cells and then shifting to a single cell type on appearance of CPE may serves as an alternate virus purification strategy. However, this will depend on the nature of the virus(es) and cell type(s) used for coinfection, if the cell line is equally permissible for both the viruses, the purification would unlikely to occur. Moreover, at higher passage, DI particle formation may interrupt the purification process.

Morphologically, antigenically and genetically confirmed clinical specimens always do not result in virus isolation in cell cultures [78,79]. Coinfection (viral interference) in our study has been found to influence the viral persistence *in vitro*, therefore, a second undesired (unknown) virus, if present, may lead to exclusion of the target virus on long-term passage and hence results in failure of isolation.

Besides documenting a natural mixed viral infection in goats, we described *in vitro* strategies for successful isolation and purification of multiple viruses. Factors such as nature of the coinfecting viruses, cell type used for coinfection, time interval of primary and secondary virus infection and, dynamics of virus replication and CPE formation determine viral interference and long-term *in vitro* copersistence and hence may influence virus isolation in the cell culture system. Further exploration of such systems may gain insights for establishing better guidelines for international trade of animals and animal products.

## Materials and Methods

### Outbreak

A severe outbreak with a mortality rate of 51.63% in goats, clinical signs resembling with those of PPR and FMD, was reported at a farmer’s goatherd at Shahjadpur, Mathura, India (December 2013). Paired saliva and nasal swabs (n = 6) from infected animals were collected after due consent from the farmer and transported to the laboratory on ice. Animals were purchased from multiple sources, about 2 months before the onset of the outbreak. No vaccination (PPR and/or FMD) was attempted by the local farmer and there was no clear vaccination history in the past.

### Identification of the viral agents in clinical specimens

The virus(es) were recovered from the clinical specimens in MEM (Sigma, Steinheim, Germany) and filtered through 0.45 μm filter. The viral RNA was extracted using QIAmp Viral RNA Mini Kit (Qiagen, Hilden, Germany). cDNA was synthesized as per the protocol described by the manufacturer (Fermentas, Hanover, USA). Briefly, 10 μl of the RNA was mixed with 200 ng of random hexamer primer and heated to 65°C for 5 min, after which it was cooled immediately on ice for 5 min and mixed subsequently with 10 μl of 5X RT buffer, 1 mM dNTPs, 40 U of RiboLock RNAse inhibitor and 200 U of Revert Aid H Minus Reverse Trasncriptase in a total reaction volume of 50 μl. The reaction mixture was incubated at 25°C for 10 min, 42°C for 1 h and 70°C for 10 min. The resulting cDNA was stored at −20°C until use.

For amplification of PPRV and FMDV-specific genome in PCR, each reaction tube of 20 μl contained 10 μl of 2X PCR master mix (Promega, Madison, USA), 20 pmol of forward and reverse primers and 2 μl of cDNA (template). The thermocycler conditions were as follows: a denaturation step of 5 min at 95°C followed by 38 cycles of amplification (30 sec at 94°C, 30 sec at 51°C, and 30 sec at 72°C), and a final extension step at 72°C for 10 min. Serotype independent amplification of FMDV genome was carried out using universal primers (forward primer: 5’-GCCTGGTCTTTCCAGGTCT-3’ and reverse primer: 5’-CAGTCCCCTTCTCAGATC-3’), that were based on conserved 5’ UTR of FMDV and have been described previously [80]. For amplification of PPRV-specific nucleoprotein (N) gene, following primers were used, forward primer: 5’- ACAGGCGCA GGTTTCATTCTT -3’ and reverse primer: 5’- GCTGAGGATATCCTTGTCGTT -3’ and has been described previously [81]. The PCR products were run in 1% agarose gel.

### Cell culture, viruses and serum

Vero cells were procured from National Centre for Cell Science (NCCS), Pune, India and were grown in MEM supplemented with 10% fetal bovine serum (FBS) (Sigma, St. Louis, USA) and antibiotics (Penicillin and streptomycin). Likewise, BHK-21 cells were also procured from NCCS, Pune and grown in Glasgov’s MEM (GMEM, Invitrogen, Bangluru, India) supplemented with 5% FBS and antibiotics (Penicillin and streptomycin, Sigma). Cocultured (BHK-21 and Vero) cells were grown in 1:1 ratio of GMEM and MEM. Vero cell adapted Newcastle disease virus (NDV) (Accession Number, VTCC-AVA155), and Buffalo poxvirus (BPXV) (Accession Number, VTCC-AVA90) available at NC-VTCC, Hisar, India were used. Anti-FMDV antibody free anti-PPRV (PPRV/Sungri/96) serum was collected from vaccinated goats at Central Institute for Research on Goats (CIRG), Mathura, India. Anti-FMDV serum (FMDV type O, A and Asia-1) from vaccinated buffalo was available in our laboratory at CIRG.

### Adaptation of the virus(es) in the cell culture

Virus recovered from the clinical specimens (filtrate) was used to infect confluent monolayers of cocultured BHK-21/Vero cells [first blind passage (BP1] and the cells were monitored daily under microscope for appearance of the CPE. Seven days following first infection, the cells were freeze-thawed twice and the resulting suspension was used to re-infect fresh coculture of BHK-21/Vero cells (BP2). The blind passages continued till appearance of the CPE.

### Purification of PPRV

To purify PPRV, primarily a plaque assay was performed in Vero cells using supernatant from the virus infected cells (BP6) as source material but no plaques could be observed. The virus (BP6) was further passaged until it produced plaques in the Vero cells at BP15. We picked up 11 plaques and tested them for both FMDV and PPRV-specific gene segments. One of the PPRV-specific plaques was further amplified to prepare a PPRV stock for further analysis.

### PPRV plaque assay

Vero cells were grown in six well plates (Corning, NY, USA) to 90–100% confluency before being infected with 500 μl of serial 10-fold dilutions of the virus in MEM for 24 h at 37°C. After removing the medium, the cells were incubated at 37°C in an agar overlay containing 13.8 g/l L-15 medium (Leibovitz, Sigma, St. Louis, USA), 15 mM HEPES (pH 7.5), 0.75 g/l sodium bicarbonate, 0.125% (w/v) bovine serum albumin (BSA), 0.05% DEAE-Dextran, 0.5% FBS, nonessential amino acids, antibiotics and 0.5% (w/v) agar. Plaques appeared by the 7^th^ day after which a second agar-overlay, additionally containing 0.03% neutral red was added and incubated for 24 h before counting the plaques.

### Characterization of plaque purified PPRV

A known anti-PPRV serum was used to neutralize viral infectivity. PPRV-specific full length M gene was also amplified by PCR using forward primer: 5'- CAGCATGGGATGTCAAAGGGTC -3' and reverse primer: 5'-CATCGTTGATGATGACATCATCGTAGACAC-3' with identical conditions described elsewhere in the manuscript except for the annealing temperature which was kept at 54°C. Gel purified PCR product was directly sequenced using forward PCR primer. The sequences were deposited in the GenBank.

### Purification of FMDV

Our attempt to purify FMDV from the virus mixture by plaque assay was unsuccessful. Since PPRV is an enveloped virus and sensitive to the organic solvents, therefore, we tried to eliminate it from the virus mixture by treating with ethanol and isopropanol (80% ethanol, 5% isopropanol) [31] by using the highest noncytotoxic concentration (1.25%). The cytotoxic concentration of the ethanol/isopropanol mixture was determined by MTT ([3-(4,5-dimethyl-2-thiazolyl)-2,5-diphenyl-2H-tetrazolium bromide] assay as described previously [82]. Similarly, hemagglutination (HA) with chicken RBCs was also performed to remove PPRV from the virus mixture. Likewise, the virus mixture at BP15 was first treated (2 h at 37°C) with anti-PPRV serum (5:1) and then infected to BHK-21 cells for 2 h at 37°C followed by washing with PBS and addition of fresh GMEM. The virus was harvested at 4–5 days post-infection by freeze thaw and tested for FMDV and PPRV-specific gene segments.

### Viral RNA transfection

Viral RNA was extracted from the mock-infected or virus mixture (BP15)-infected cells using QIAmp Viral RNA Mini Kit (Qiagen, Hilden, Germany) as described elsewhere in the manuscript. BHK-21 cells were grown in 12 well tissue culture plates and when the monolayer was ~ 80% confluent it was transfected with 250 ng of RNA using Lipofectamine® MessengerMAX™ Transfection Reagent (Invitrogen, Carlsbad, USA) as per the protocol described by the manufacturer. At 6 hpt, the cells were washed three times with PBS followed by addition of fresh GMEM. Cells were observed daily for appearance of the CPE. At 48 hpt, the infected cell culture supernatant was collected and evaluated for PPRV and FMDV-specific gene segments by PCR.

### FMDV infection

Purified FMDV was amplified and titrated in BHK-21 cells. Briefly, ~ 90% confluent monolayers of BHK-21 cells were infected with FMDV (unless otherwise indicated, at MOI = 1) for 1h at 37°C followed by washing three times with PBS and addition of fresh GMEM (supplanted with 1% FBS). The supernatant was harvested when > 50% cells showed the CPE. The virus was titrated in BHK-21 cells by determining TCID_50_ at two-fold serial dilutions.

### FMDV plaque assay

FMDV plaque assay was performed by adopting some modifications from the existing protocols [28–30]. Briefly, BHK-21 cells were grown in six well plates to 90–100% confluency before being infected with 500 μl of serial 10-fold dilutions of the virus in GMEM for 45 min at 37°C. After removing the medium, the cells were incubated at 37°C in a similar agar-overlay described elsewhere in the manuscript. Plaques appeared within 48 hpi after which the agar-overlay was removed and plates were stained with crystal violet.

### FMDV serotyping

FMDV serotyping was performed by PCR using serotype specific primers that have been previously described [83]. PCR products, amplified using serotype independent (5 ‘UTR) and serotype-specific (FMDV type-O) primers, were directly sequenced and the resulting sequences were deposited to the GenBank.

### Long-term coinfections

Vero cells were coinfected with combination of viruses viz: FMDV+PPRV, FMDV+NDV, FMDV+BPXV, PPRV+ NDV, PPRV+BPXV, NDV+BPXV, each at MOI of 1 for 2 h followed by washing 5 times with PBS and addition of fresh MEM. Virus was harvested when >50% cells showed the CPE. If there was no CPE, the cells were cultured for 8–10 days and freeze-thawed to harvest the virus. 200 μl of the cell culture supernatant from the first passage was used to infect fresh cells for the next passage. Five such passages were made. Viral RNA/DNA Purification Kit (Thermoscientific, Vilnius, Lithunia) was used for simultaneous extraction of both viral RNA and DNA from coinfected cell culture supernatants. The primer sequence for detection of NDV (Fusion gene) and BPXV (C18L gene) were as follows: NDV; forward primer: 5’- GTCTACCAGGCATTCGCTTCTTCTACCAGGATCCCAGCAC -3’ and reverse primer: 5’- TGGGTGACTCAATTCTGCTGATGCCTCTAATGGGGCTTT -3’ and BPXV; forward primer: 5’- GCGGGTATCACTGTTATGAAACC-3’ and reverse primer: 5’-CATAAATACACTTTTATAGTCCTCG-3’. Primers used for amplification of PPRV and FMDV have been described elsewhere in the manuscript. PCR conditions were identical as described elsewhere in the manuscript except annealing temperature which was kept at 55°C for both for NDV and BPXV.

### qRT-PCR

The levels of viral RNA in the infected cell culture supernatant were quantified by qRT-PCR. Viral RNA extraction and cDNA synthesis was performed as described above. qRT-PCR was carried out with a 20 μl reaction mixture containing gene specific primers, template and Sybr green DNA dye (Promega, Madison, USA) and run on CFX96 RealTime PCR detection system (Bio-Rad, USA). The primers used for qRT-PCR were same as described above for diagnostic PCR. Thermalcycler conditions were as follows: a denaturation step of 5 min at 94°C followed by 40 cycles of amplification (30 s at 94°C, 30 s at 51°C, and 30 s at 72°C). The viral RNA levels, expressed as threshold cycle (*CT*) values, were analyzed to determine relative fold change in RNA copy number as described previously [84].
